# The correlation between systemic immune-inflammation index and major depression in patients with depression

**DOI:** 10.3389/fpsyt.2025.1580151

**Published:** 2025-05-08

**Authors:** Yixuan Bai, Zanxi Fang, Huirong Dai, Qiao Zhang, Pan You

**Affiliations:** ^1^ Department of Clinical Laboratory, Xiamen Xianyue Hospital, Xianyue Hospital Affiliated with Xiamen Medical College, Fujian Psychiatric Center, Fujian Clinical Research Center for Mental Disorders, Xiamen, Fujian, China; ^2^ Centre of Clinical Laboratory, Zhongshan Hospital of Xiamen University, School of Medicine, Xiamen University, Xiamen, Fujian, China

**Keywords:** systemic immune-inflammatory index, depression, severity, major depression, correlation

## Abstract

**Aims:**

To explore the relationship between the systemic immune-inflammation index (SII) and the severity of depression.

**Methods:**

This retrospective study included 750 patients who were hospitalized at Xiamen Xianyue Hospital and diagnosed with depression from January 2022 to December 2023. The SII was defined as the platelet count × neutrophil count/lymphocyte count. The participants were divided into a mild to moderate depression group (299 patients) and a major depression group (451 patients). Univariate and multivariate Logistic regression analysis, subgroup analysis, sensitivity analysis, and receiver operating characteristic (ROC) curve analysis were used to explore the correlation between the SII and the severity of depression.

**Results:**

According to the multivariate Logistic regression analysis, the SII was independently associated with the risk of major depression (P < 0.05). For every 1- unit and 1-standard-deviation increase in the SII, the risk of major depression increased by 0.1% and 25.3%, respectively (OR: 1.001, 95% CI: 1.000–1.001, P = 0.008; OR: 1.253, 95% CI: 1.061–1.480, P = 0.008), and each 1-unit increase in the Log_10_SII was associated with a 124.8% increased risk of major depression (OR: 2.248, 95% CI: 1.231–4.106, P = 0.008). Subgroup analysis and sensitivity analysis revealed significant associations between the SII and the risk of major depression was significant in multiple specific populations (P < 0.05). ROC curve analysis revealed that the area under the curve (AUC) value for using the SII to predict the risk of major depression was 0.585 (95% CI: 0.507–0.591, P = 0.024).

**Conclusion:**

Higher SII values are strongly associated with a greater risk of major depression.

## 1 Introduction

Depression is one of the leading mental illnesses in the world and is characterized by persistent low mood and loss of interest in almost all activities, accompanied by symptoms such as sleep disturbances, fatigue, anxiety and neurocognitive impairment, which reduce the quality of life for many people (1). According to the World Health Organization, approximately 5% of the global population suffers from depression (2). Depression constitutes a significant economic burden on the national health system, and it not only affects the lives of patients through reduced working ability, loss of social function, and increased risk of alcohol and drug abuse but is also associated with a greater risk of suicide (3, 4). Globally, depression, as measured by disability-adjusted life years, is projected to be the leading contributor to the global burden of disease by 2030 (5).

Depression is the result of the interaction of multiple factors, and its etiology is complex and not fully understood (6). Studies have shown that inflammatory processes are related to the pathophysiology of depression, and have described how the immune system regulates mood and the potential causes of dysregulated inflammatory response in depressed patients (7). Microglia in the central nervous system, which respond to stress-induced neuroinflammation, play an important role in the development and progression of depression by releasing proinflammatory cytokines and their metabolites (8). Studies have also revealed that the levels of various inflammatory factors including C-reactive protein (CRP), interleukin-6 (IL-6), interleukin-12 (IL-12), interleukin-18 (IL-18), soluble IL-2 receptor and tumor necrosis factor-α (TNF-α) are significantly greater in depressed patients than in healthy controls (9, 10). In addition, regulatory T cells, eosinophils and other immune cells play key roles in the development of depression (11, 12). Therefore, it is necessary to investigate the correlations of inflammatory status with depression risk and severity.

Current studies have shown that as an emerging medical index used to comprehensively reflect systemic inflammation and immune status, the systemic immune-inflammation index (SII) has become a popular research topic related to inflammation (13). For example, the level of SII is often elevated in patients with malignant tumors such as prostate cancer and colorectal cancer, indicating that although the SII cannot be used as a diagnostic criterion for tumors alone, it has certain predictive value for tumor recurrence (14, 15). In addition, the SII is also associated with the severity of infectious diseases such as COVID-19 and is closely associated with the risk of death from sepsis (16, 17). In addition, the SII is also valuable in the diagnosis, prognosis assessment and treatment monitoring of autoimmune diseases such as systemic lupus erythematosus (18). More recently, the SII has been linked to a variety of mental illnesses. For example, patients with bipolar disorder had a significantly higher SII index than healthy controls did, suggesting higher levels of inflammation in such patients (19). In addition, Li et al. revealed the relationship between the SII and cognitive decline in Alzheimer’s disease patients (20), and Steiner et al. reported that the improvement in positive symptoms after treatment for schizophrenia was associated with a decrease in neutrophil count, suggesting the neutrophil count may be a useful marker of schizophrenia severity and treatment response (21). In general, stroke can lead to a variety of mental disorders, such as depression, and a positive correlation between the SII level and post-stroke depression has also been confirmed (22). However, the correlation between the SII and depression severity is unclear. Therefore, to fill this knowledge gap, we conducted this cross-sectional study to assess the relationship between the SII and the risk of major depression, providing new clues and a theoretical basis for exploring the SII in depression diagnosis and treatment of depression and risk assessment.

## 2 Methods

### 2.1 Study population

In this single-center, prospective, retrospective, observational, clinical study, all participants were patients with depression who were hospitalized at Xiamen Xianyue Hospital between January 2022 and December 2023. After excluding patients under 30 years of age and those without platelet count, neutrophil count, lymphocyte count, or depression evaluation data, 750 patients were enrolled in the study. The research protocol was approved by the Ethics Committee of Xiamen Xianyue Hospital and conformed to the basic principles of the Helsinki Declaration. Owing to the retrospective nature of this study, patients’ informed consent was waived.

### 2.2 Variable collection and definition

Demographic variables, anthropometric variables, comorbidities and medication variables, and biomarker variables were included in the study. The demographic variables included age, sex, family history of depression, and smoking. The anthropometric variables included height, weight, body mass index (BMI), systolic blood pressure (SBP), diastolic blood pressure (DBP), and heart rate, with BMI defined as weight (kg)/height (m^2^). The comorbidities and drug treatment variables included diabetes status, hypertension status, coronary heart disease (CHD) status, ischemic stroke status, hypotensive drug use, hypoglycemic drugs use, and antiagglomerant use. Diabetes was defined as having a history of diabetes or being treated with hypoglycemic drugs, fasting blood glucose (FBG) ≥ 7.0 mmol/L or glycosylated hemoglobin (HbA1c) ≥ 6.5% (23). Hypertension was defined as having a history of hypertension or being treated with hypotensive drugs or SBP/DBP ≥ 140/90 mmHg (24). CHD was defined as a history of CHD. Ischemic stroke was defined as a history of prior ischemic stroke. The biomarker variables included FBG, triglycerides, total cholesterol, low density lipoprotein cholesterol (LDL-C), high density lipoprotein cholesterol (HDL-C), apolipoprotein A1, apolipoprotein B, albumin, uric acid, urea nitrogen, creatinine, white blood cell (WBC)counts, neutrophil counts, lymphocyte counts, monocyte counts, hemoglobin, and platelet counts. All blood markers were measured in venous blood from patients who had fasted for at least 8 hours; the samples were sent to a standard laboratory and assessed via standardized testing methods.

### 2.3 Assessment of the SII and depression status

In this study, the SII was calculated as platelet count × neutrophil count/lymphocyte count (14). Patients were divided into three groups based on the SII tertiles: low SII (n = 250), medium SII (n = 250), and high SII (n = 250). According to the International Classification of Diseases-10 criteria, the diagnostic criteria for depression are divided into typical symptoms and other common symptoms. Typical symptoms include low mood, loss of interest and pleasure, low energy or fatigue. Other common symptoms include reduced ability to focus and pay attention, reduced self-evaluation, feelings of self-guilt and worthlessness, a bleak outlook, pessimism, thoughts or behaviors of self-harm or suicide, sleep disturbances, and decreased appetite (25). The course of depressive disorder lasts at least two weeks. According to its severity, depressive episodes are divided into the following: 1) mild depression, with at least two typical symptoms and at least two other symptoms, and the patient has certain difficulties in daily work and social activities, which has an impact on the patient’s social function; 2) moderate depression, with at least two typical symptoms and at least three other symptoms, and the patient has considerable difficulty with work, social, or household activities; and 3) major depression, with all three typical symptoms present and at least four other symptoms, and when the symptoms are extremely severe or the onset of the illness is very rapid, the diagnosis can be made in less than two weeks: except for a very limited extent, it is almost impossible for the patient to socialize, work, or do housework (26). In this study, depression was divided into two groups on the basis of severity: mild to moderate depression (n = 299) and major depression (n = 451).

### 2.4 Statistical methods

All the statistical tests were conducted using SPSS 26.0. Continuous variables conforming to a normal distribution are presented as the means ± standard deviations, and differences between the two and three groups were evaluated by independent sample T-test or one-way ANOVA, respectively. Continuous variables that did not conform to the normal distribution were represented by the median (quartiles), and differences between the two and three groups were assessed by non-parametric tests. Categorical variables are expressed as frequencies (percentages), and differences between groups were evaluated via the chi-square test. Univariate Logistic regression analysis was used to assess the predictors of major depression, and multivariate Logistic regression analysis was performed to assess the independent association between the SII and major depression. Subgroup analyses were conducted to further explore the stratified associations between the SII and major depression by age, sex, hypertension, and BMI. A sensitivity analysis was performed by excluding patients with CHD and ischemic stroke, and the reliability of the association between the SII and major depression was again validated via multivariate Logistic regression analysis. Finally, the predictive value of the SII for the risk of major depression was evaluated using receiver operating characteristic (ROC)curve analysis. All tests were two-sided, and a P value < 0.05 was considered to indicate statistical significance.

## 3 Results

### 3.1 Baseline characteristics

As shown in **Table 1**, a total of 750 patients with depression were included in this study, with an average age (51.65 ± 12.99) years; 223 males were included (29.7%). Compared with mild-moderate depression patients, major depression patients were younger, more likely to be male, less likely to use hypotensive drugs, and had higher neutrophil counts, monocyte counts, SIIs, Standardized SIIs and Log_10_SIIs (P < 0.05).

**Table 1:** Baseline characteristics of groups based on depression severity.

Variables	Total population	Mild to moderate depression	Major depression	P value
N	750	299	451	
Age, year	51.65 ± 12.99	54.53 ± 12.22	49.75 ± 13.14	< 0.001
Male, n (%)	223 (29.7)	74 (24.7)	149 (33.0)	0.015
Family history, n (%)	69 (9.2)	28 (9.4)	41 (9.1)	0.899
Smoking, n (%)	68 (9.1)	25 (8.4)	43 (9.5)	0.584
Diabetes, n (%)	70 (9.3)	29 (9.7)	41 (9.1)	0.787
Hypertension, n (%)	205 (27.3)	93 (31.1)	112 (24.8)	0.059
Coronary heart disease, n (%)	14 (1.9)	6 (2.0)	8 (1.8)	0.818
Ischemic stroke, n (%)	11 (1.5)	7 (2.3)	4 (0.9)	0.105
Hypotensive drugs, n (%)	123 (16.4)	60 (20.1)	63 (14.0)	0.027
Hypoglycemic drugs, n (%)	46 (6.1)	20 (6.7)	26 (5.8)	0.606
Antiagglomerant, n (%)	19 (2.5)	8 (2.7)	11 (2.4)	0.840
BMI, kg/m^2^	22.58 ± 3.66	22.84 ± 3.18	22.41 ± 3.94	0.122
SBP, mmHg	122.80 ± 16.27	123.15 ± 15.51	122.58 ± 16.77	0.637
DBP, mmHg	80.22 ± 10.41	80.03 ± 10.87	80.35 ± 10.10	0.684
HR, bpm	86.68 ± 42.71	85.78 ± 42.33	87.27 ± 43.00	0.639
FPG, mmol/L	5.19 ± 1.01	5.20 ± 0.99	5.18 ± 1.03	0.815
Triglycerides, mmol/L	1.22 (0.90, 1.80)	1.29 (0.89, 1.93)	1.19 (0.90, 1.68)	0.090
Total cholesterol, mmol/L	5.02 ± 1.06	5.06 ± 1.09	5.00 ± 1.09	0.420
LDL-C, mmol/L	3.10 ± 0.83	3.11 ± 0.86	3.09 ± 0.82	0.754
HDL-C, mmol/L	1.32 ± 0.35	1.33 ± 0.37	1.32 ± 0.33	0.754
ApoA1, g/L	1.27 ± 0.30	1.28 ± 0.30	1.26 ± 0.29	0.495
ApoB, g/L	0.93 ± 0.27	0.94 ± 0.28	0.93 ± 0.26	0.622
Albumin, g/L	41.45 ± 3.48	41.41 ± 3.50	41.47 ± 3.47	0.824
Uric acid, ųmol/L	299.61 ± 82.56	295.24 ± 79.30	302.50 ± 84.61	0.238
Urea nitrogen, mmol/L	4.67 ± 1.43	4.66 ± 1.26	4.66 ± 1.53	0.981
Creatinine, ųmol/L	64.62 ± 13.62	63.74 ± 12.93	65.20 ± 12.93	0.142
WBC, x10^9^/L	6.40 ± 1.83	6.27 ± 1.66	6.48 ± 1.93	0.111
Neutrophil counts, x10^9^/L	3.77 ± 1.52	3.63 ± 1.37	3.87 ± 1.61	0.028
Lymphocyte counts, x10^9^/L	1.98 ± 0.62	2.02 ± 0.61	1.96 ± 0.63	0.205
Monocyte counts, x10^9^/L	0.47 ± 0.18	0.46 ± 0.16	0.49 ± 0.19	0.037
Hemoglobin, g/L	131.13 ± 14.86	130.86 ± 15.05	131.81 ± 15.05	0.688
Platelets, x10^9^/L	248.03 ± 63.63	246.07 ± 60.93	249.33 ± 65.39	0.492
SII, x10^9^/L	428.91 (307.84, 646.45)	411.39 (295.09, 609.67)	450.93 (315.36, 668.10)	0.024
Standardized SII	-0.28 (-0.62, 0.34)	-0.33 (-0.66, 0.24)	-0.21(0.59, 0.40)	0.024
Log_10_SII	2.65 ± 0.25	2.62 ± 0.24	2.66 ± 0.26	0.029

As shown in **Table 2**, there were significant differences in the prevalence of diabetes and hypertension, the rates of hypotensive drug use, SBP, DBP, FBG, creatinine, WBC counts, neutrophil counts, lymphocyte counts, monocyte counts, hemoglobin, and platelet counts and the prevalence of major depression among the three SII groups (P < 0.05).

**Table 2:** Baseline characteristics grouped by SII tertiles.

Variable	Low SII	Medium SII	High SII	P value
N	250	250	250	
Age, year	50.58 ± 12.85	51.65 ± 12.75	52.73 ± 13.32	0.180
Male, n (%)	74 (29.6)	81 (32.4)	68 (27.2)	0.445
Family history, n (%)	22 (8.8)	27 (10.8)	20 (8.0)	0.537
Smoking, n (%)	16 (6.4)	31 (12.4)	21 (8.4)	0.058
Diabetes, n (%)	16 (6.4)	17 (6.8)	37 (14.8)	0.001
Hypertension, n (%)	47 (18.8)	70 (28.0)	88 (35.2)	< 0.001
Coronary heart disease, n (%)	2 (0.8)	5 (2.0)	7 (2.8)	0.099
Ischemic stroke, n (%)	7 (2.8)	1 (0.4)	3 (1.2)	0.070
Hypotensive drugs, n (%)	25 (10.0)	43 (17.2)	55 (22.0)	<0.001
Hypoglycemic drugs, n (%)	12 (4.8)	13 (5.2)	21 (8.4)	0.094
Antiagglomerant, n (%)	7 (2.8)	4 (1.6)	8 (3.2)	0.473
BMI, kg/m^2^	51.65 ± 12.99	123.53 ± 12.22	49.75 ± 13.14	0.451
SBP, mmHg	119.45 ± 15.30	123.99 ± 16.32	124.97 ± 16.70	< 0.001
DBP, mmHg	78.64 ± 9.88	80.85 ± 10.65	81.17 ± 10.41	0.013
HR, bpm	82.08 ± 11.12	87.91 ± 45.93	90.05 ± 56.75	0.097
FPG, mmol/L	5.02 ± 0.81	5.17 ± 0.95	5.38 ± 1.20	< 0.001
Triglycerides, mmol/L	1.27 (0.89, 1.80)	1.24 (0.91, 1.85)	1.19 (0.91, 1.72)	0.839
Total cholesterol, mmol/L	5.06 ± 1.06	5.03 ± 1.05	4.98 ± 1.06	0.716
LDL-C, mmol/L	3.15 ± 0.83	3.08 ± 0.85	3.07 ± 0.84	0.523
HDL-C, mmol/L	1.29 ± 0.35	1.35 ± 0.35	1.32 ± 0.34	0.162
ApoA1, g/L	1.23 ± 0.28	1.29 ± 0.31	1.27 ± 0.30	0.058
ApoB, g/L	0.93 ± 0.27	0.93 ± 0.27	0.94 ± 0.27	0.975
Albumin, g/L	41.27 ± 3.28	41.67 ± 3.09	41.40 ± 3.99	0.434
Uric acid, umol/L	306.18 ± 82.30	301.94 ± 86.77	290.70 ± 77.91	0.096
Urea nitrogen, mmol/L	4.68 ± 1.32	4.72 ± 1.40	4.60 ± 1.56	0.626
Creatinine, umol/L	64.70 ± 13.02	66.28 ± 14.94	62.88 ± 12.93	0.020
WBC, x10^9^/L	5.41 ± 1.39	6.36 ± 1.54	7.41 ± 1.93	< 0.001
Neutrophil counts, x10^9^/L	2.63 ± 0.82	3.65 ± 0.94	5.03 ± 1.59	< 0.001
Lymphocyte counts, x10^9^/L	2.21 ± 0.66	2.05 ± 0.60	1.69 ± 0.48	< 0.001
Monocyte counts, x10^9^/L	0.41 ± 0.14	0.48 ± 0.17	0.54 ± 0.19	< 0.001
Hemoglobin, g/L	131.26 ± 13.98	133.03 ± 15.03	129.10 ± 15.35	0.012
Platelets, x10^9^/L	210.13 ± 45.42	247.41 ± 49.54	286.56 ± 68.76	< 0.001
Depression, n (%)				0.022
Mild to Moderate	113 (45.2)	98 (39.2)	88 (35.2)	
Major	137 (54.8)	152 (60.8)	162 (64.8)	

### 3.2 Associations of other covariates with the SII and depression severity

The results of the Spearman correlation analysis presented in **Table 3** revealed that the SII was positively correlated with diabetes status, hypertension status, CHD status, hypotensive drug use, hypoglycemic drug use, SBP, DBP, heart rate, FBG levels, WBC levels, the neutrophil count, the monocyte count and major depression status, but negatively correlated with uric acid levels and the lymphocyte count (P < 0.05). In addition, major depression was negatively correlated with age, female sex, hypotensive drug use and BMI, but positively correlated with the SII (P < 0.05).

**Table 3:** Associations of other covariates with SII and depression severity.

Variable	SII		Major Depression	
	r	P	r	P
Age	0.069	0.059	-0.183	< 0.001
Male	0.015	0.688	0.089	0.015
Family history	-0.005	0.890	-0.005	0.899
Smoking	0.041	0.263	0.020	0.584
Diabetes	0.136	< 0.001	-0.010	0.787
Hypertension	0.151	< 0.001	-0.069	0.059
Coronary heart disease	0.077	0.035	-0.008	0.818
Ischemic stroke	-0.058	0.113	-0.059	0.105
Hypotensive drugs	0.135	< 0.001	-0.081	0.027
Hypoglycemic drugs	0.075	0.040	-0.019	0.606
Antiagglomerant	0.022	0.540	-0.007	0.840
BMI	-0.017	0.640	-0.078	0.033
SBP	0.151	< 0.001	-0.017	0.638
DBP	0.082	0.024	0.014	0.696
HR	0.157	< 0.001	0.063	0.086
FPG	0.143	< 0.001	-0.028	0.440
Triglycerides	0.021	0.564	-0.062	0.090
Total cholesterol	-0.023	0.534	-0.031	0.403
LDL-C	0.035	0.336	-0.008	0.833
HDL-C	0.006	0.867	-0.001	0.750
ApoA1	0.036	0.324	-0.030	0.412
ApoB	0.023	0.536	-0.009	0.810
Albumin	0.015	0.676	0.017	0.650
Uric acid	-0.073	0.044	0.031	0.397
Urea nitrogen	-0.042	0.251	-0.027	0.453
Creatinine	-0.068	0.064	0.050	0.173
WBC	0.495	< 0.001	0.038	0.294
Neutrophil counts	0.743	< 0.001	0.063	0.082
Lymphocyte counts	-0.372	< 0.001	-0.059	0.104
Monocyte counts	0.301	< 0.001	0.069	0.060
Hemoglobin	-0.039	0.286	0.033	0.364
Platelets	0.554	< 0.001	0.009	0.808
SII	–	–	0.083	0.024
Major depression	0.083	0.024	–	–

### 3.3 Association of SII with depression severity

As shown in **Table 4**, univariate Logistic regression analysis revealed that age, male sex, hypotensive drug use, neutrophil count, monocyte count and the SII were closely correlated with major depression (P < 0.05), whereas hypertension and triglycerides were weakly correlated with major depression (0.05 < P < 0.01). Then, variables with P < 0.1 in the univariate Logistic regression analysis were included in the multivariate Logistic regression analysis and three models were constructed to gradually explore the correlation between the SII and major depression (**Table 5**). In unadjusted Model 1 (unadjusted) and Model 2 (adjusted only for age and sex), the SII was strongly associated with the risk of major depression regardless of whether it was used as a categorical variable or a continuous variable (P < 0.05). In Model 3, which was further adjusted for hypertension status, hypotensive drug use, triglyceride levels, neutrophil counts and monocyte counts on the basis of Model 2, the SII was still independently associated with the risk of major depression (P < 0.05). For every 1-unit and 1-standard-deviation increase in the SII, the risk of major depression increased by 0.1% and 25.3%, respectively (OR: 1.001, 95% CI: 1.000-1.001, P = 0.008; OR: 1.253, 95% CI: 1.061–1.480, P = 0.008), and each 1-unit increase in Log_10_SII was associated with a 124.8% increased risk of major depressive (OR: 2.248, 95% CI:1.231–4.106, P = 0.008).

**Table 4:** Univariate Logistic regression analysis of depression severity.

Variable	OR	95% CI	P value
Age	0.971	0.960-0.983	< 0.001
Male	1.500	1.081-2.082	0.015
Family history	1.033	0.624-1.711	0.899
Smoking	0.866	0.517-1.451	0.584
Diabetes	1.071	0.650-1.766	0.787
Hypertension	1.366	0.987-1.891	0.060
Coronary heart disease	1.134	0.389-3.302	0.818
Ischemic stroke	2.679	0.777-9.232	0.119
Hypotensive drugs	1.546	1.048-2.280	0.028
Hypoglycemic drugs	1.172	0.642-2.140	0.606
Antiagglomerant	1.100	0.437-2.767	0.840
BMI	0.969	0.930-1.009	0.127
SBP, mmHg	0.998	0.989-1.007	0.636
DBP, mmHg	1.003	0.989-1.017	0.684
HR	1.001	0.997-1.005	0.645
FPG	0.983	0.851-1.135	0.815
Triglycerides	0.836	0.696-1.005	0.057
Total cholesterol	0.945	0.823-1.085	0.420
LDL-C	0.973	0.817-1.158	0.754
HDL-C	0.935	0.613-1.425	0.754
ApoA1	0.843	0.516-1.376	0.495
ApoB	0.873	0.508-1.498	0.244
Albumin	1.005	0.963-1.048	0.824
Uric acid	1.001	0.999-1.003	0.238
Urea nitrogen	1.001	0.904-1.109	0.982
Creatinine	1.008	0.997-1.019	0.149
WBC	1.066	0.983-1.156	0.122
Neutrophil counts	1.113	1.008-1.229	0.035
Lymphocyte counts	0.859	0.680-1.087	0.205
Monocyte counts	2.475	1.051-5.831	0.038
Hemoglobin	1.002	0.992-1.012	0.688
Platelets	1.001	0.998-1.003	0.492
SII	1.001	1.000-1.001	0.023
Standardized SII	1.205	1.025-1.415	0.023
Log_10_SII	1.917	1.066-3.447	0.030

**Table 5:** Multivariate Logistic regression analysis of SII and depression severity.

Variable	Model 1			Model 2			Model 3		
	OR	95% CI	P value	OR	95% CI	P value	OR	95% CI	P value
SII (Categorical variable)									
Low SII	Ref			Ref					
Medium SII	1.729	0.896-1.826	0.175	1.320	0.917-1.900	0.135	1.284	0.869-1.898	0.209
High SII	1.518	1.060-2.176	0.023	1.657	1.145-2.397	0.007	1.540	0.940-2.523	0.087
SII (Continuous variable)									
SII	1.001	1.000-1.001	0.023	1.001	1.000-1.001	0.008	1.001	1.000-1.001	0.008
Standardized SII	1.205	1.025-1.415	0.023	1.253	1.061-1.480	0.008	1.253	1.061-1.480	0.008
Log_10_SII	1.917	1.066-3.447	0.030	2.248	1.231-4.106	0.008	2.248	1.231-4.106	0.008

Model 1: Unadjusted; Model 2: Adjusting for age and sex; Model 3: Adjusting for age, sex, hypertension, hypotensive drugs, triglycerides, neutrophil counts, and Monocyte counts.

### 3.4 Stratified association of the SII with major depression

As shown in **Table 6**, for every 1-unit and 1-standard-deviation increase in the SII in women, the risk of major depressive increased by 0.1% and 21.7%, respectively (OR: 1.001, 95% CI: 1.000–1.001, P = 0.046; OR: 1.217, 95% CI: 1.003–1.475, P = 0.046). Among people aged 50 years or older, the risk of major depression was greater in the high SII group than in the low SII group (OR: 1.976, 95% CI: 1.021–3.823, P =0.043). In people without hypertension, each 1-unit increase in the Log_10_SII was associated with a 395.6% increased risk of major depressive (OR: 4.956, 95% CI: 1.375–17.868, P = 0.014). Among those with a BMI ≥ 24 kg/m^2^, the risk of major depression was lower in the moderate SII group than in the low SII group (OR: 0.369, 95% CI:0.142–0.958, P = 0.040).

**Table 6:** Stratified association of SII with depression severity.

Variable	SII as categorical variable						SII as continuous variable					
	Low SII		Medium SII		High SII		SII		Standardized SII		Log_10_SII	
	OR (95% CI)	P value	OR (95% CI)	P value	OR (95% CI)	P value	OR (95% CI)	P value	OR (95% CI)	P value	OR (95% CI)	P value
Sex												
Male	Ref		0.545 (0.197-1.507)	0.242	0.504 (0.219-1.160)	0.107	1.001 (0.999-1.002)	0.430	1.196 (0.767-1.866)	0.430	2.720 (0.448-16.515)	0.277
Female	Ref		0.719 (0.404-1.278)	0.260	1.015 (0.618-1.665)	0.954	1.001 (1.000-1.001)	0.046	1.217 (1.003-1.475)	0.046	1.857 (0.628-5.487)	0.263
Age												
30–50 years	Ref		1.100 (0.518-2.337)	0.804	1.193 (0.615-2.313)	0.602	1.001 (0.999-1.001)	0.530	1.129 (0.773-1.649)	0.530	1.253 (0.294-5.350)	0.760
≥ 50 years	Ref		1.443 (0.863-2.413)	0.162	1.976 (1.021-3.823)	0.043	1.001 (1.000-1.001)	0.194	1.234 (0.898-1.694)	0.194	2.245 (0.680-7.418)	0.185
Hypertension												
Yes	Ref		0.403 (0.155-1.046)	0.062	0.533 (0.257-1.102)	0.090	1.001 (1.000-1.002)	0.252	1.260 (0.848-1.873)	0.252	1.822 (0.617-5.375)	0.277
No	Ref		0.776 (0.430-1.402)	0.401	1.027 (0.611-1.726)	0.921	1.001 (1.000-1.002)	0.141	1.278 (0.922-1.773)	0.141	4.956 (1.375-17.868)	0.014
BMI												
< 24 kg/m^2^	Ref		0.825 (0.457-1.488)	0.825	0.968 (0.585-1.603)	0.901	1.000 (1.000-1.001)	0.363	1.136 (0.864-1.494)	0.363	1.653 (0.559-4.888)	0.364
≥ 24 kg/m^2^	Ref		0.369 (0.142-0.958)	0.040	0.621 (0.285-1.353)	0.230	1.001 (1.000-1.003)	0.072	1.613 (0.958-2.715)	0.072	4.132 (0.674-25.315)	0.125

### 3.5 Sensitivity analysis

As shown in **Table 7**, multivariate Logistic regression analysis was performed after excluding CHD and ischemic stroke patients. In unadjusted Model 1 and Model 2 adjusted for only age and sex, the SII was strongly associated with the risk of major depressive (P < 0.05), regardless of whether the SII was used as a categorical variable or a continuous variable. In Model 3, adjusted for age, sex, hypertension status, hypotensive drug use, triglyceride levels, neutrophil counts and monocyte counts, the SII was still independently associated with the risk of major depression (P < 0.05). For every 1-unit and 1-standard-deviation increase in the SII, the risk of major depressive increased by 0.1% and 25.6%, respectively (OR: 1.001, 95% CI: 1.000–1.001, P = 0.009; OR: 1.256, 95% CI: 1.059–1.490, P = 0.009), and each 1-unit increase in the Log_10_SII was associated with a 128.6% increased risk of major depression (OR: 2.286, 95% CI: 1.236–4.229, P = 0.008), and the risk of major depression was increased by 64.8% in the high SII group compared with the low SII group (OR: 1.648, 95% CI: 1.132–2.399, P = 0.009).

**Table 7:** Multivariate Logistic regression analysis of SII and depression severity: excluding patients with coronary heart disease and ischemic stroke.

Variable	Model 1			Model 2			Model 3		
	OR	95% CI	P value	OR	95% CI	P value	OR	95% CI	P value
SII (Categorical variable)									
Low SII	Ref			Ref					
Medium SII	1.331	0.927-1.911	0.175	1.378	0.952-1.996	0.090	1.378	0.952-1.996	0.090
High SII	1.522	1.055-2.196	0.025	1.648	1.132-2.399	0.009	1.648	1.132-2.399	0.009
SII (Continuous variable)									
SII	1.001	1.000-1.001	0.023	1.001	1.000-1.001	0.009	1.001	1.000-1.001	0.009
Standardized SII	1.213	1.027-1.431	0.023	1.256	1.059-1.490	0.009	1.256	1.059-1.490	0.009
Log_10_SII	1.970	1.082-3.588	0.027	2.286	1.236-4.229	0.008	2.286	1.236-4.229	0.008

Model 1: Unadjusted; Model 2: Adjusting for age and sex; Model 3: Adjusting for age, sex, hypertension, hypotensive drugs, triglycerides, neutrophil counts, and Monocyte counts.

### 3.6 The value of the SII for predicting major depression

As shown in **Supplementary Figure 1**, the ROC curve revealed that the area under the curve (AUC) value used by the SII to predict the risk of major depression was 0.585 (95% CI: 0.507–0.591, P = 0.024).

## 4 Discussion

In this single-center cross-sectional observational study from China, we not only demonstrated an independent association of the SII with the risk of major depression. Moreover, a strong association between higher levels of SII values and a greater risk of major depression was also found in several subgroups, including females, those aged ≥ 50 years, those without hypertension, and those with a BMI > 42 kg/m^2^. In addition, after excluding patients with CHD and ischemic stroke, we also found that the SII was independently associated with the risk of major depressive, whether as a categorical or continuous variable. Finally, ROC curve analysis also revealed that the SII has a certain predictive value for the occurrence of major depression. These data suggest that the SII can be incorporated into the daily management of early depression screening and risk assessment.

There is increasing evidence that inflammation is related not only to the occurrence and development of cardiovascular and cerebrovascular diseases, metabolic diseases and tumors, but also to the risk of developing mental diseases. For example, Mohd Asyraf et al. reported that compared with healthy controls, patients with schizophrenia had higher levels of peripheral complement C3 and C4, and complement C3 and C4 are potential peripheral biomarkers for schizophrenia, which indicates that the role of inflammatory indicators in the diagnosis of mental diseases should be emphasized (27). In addition, Fusar-Poli et al. found through a cross-sectional study involving 294 patients with bipolar affective disorder that there were significant differences in neutrophils among patients with bipolar affective disorder in different emotional episode states, and the platelet-to-lymphocyte ratio could be regarded as a unique and independent predictor of (hypo) mania, which indicates that the inflammatory system plays an important role in the occurrence and progression of mood disorders (28). Depression is also an important mental disorder. Ng et al. included 34 related studies in a meta-analysis and reported that the level of IL-6 in peripheral blood was significantly increased in elderly people with depression; thus, elderly patients should be monitored for excessive IL-6 levels during daily management (29). Furthermore, a prospective birth cohort reported that those with an increase in CRP from adolescence to early adulthood had a greater risk of developing moderate/severe depression at the age of 18, which suggests that we should pay attention to the increase in CRP levels among adolescents to assess the risk of developing depression (30). All the above studies revealed correlations between inflammatory indicators and depression.

Recently, the SII, as an emerging inflammatory indicator, has attracted attention because of its low cost and convenient detection. Some studies based on data from the National Health and Nutrition Examination Survey (NHANES) revealed that for every one-unit increase in log_2_-SII, the incidence of depression increases by 18% (31). On the other hand, an increase in the SII has also been confirmed to be a predictor of depression and anxiety development in patients with tuberculosis and COVID-19 (32, 33). However, the current research on SII in patients with depression is insufficient. To our knowledge, few studies have explored the correlation between the SII and the severity of depression. Zhu et al. reported that there were differences in the SII levels between patients with depression and healthy controls, but there was no statistically significant difference in the SII among different subgroups of depression in the 239 patients included in the study (34). Putrada et al. showed that the SII was superior to monocyte-to-lymphocyte ratio (MLR), platelet-to-lymphocyte ratio (PLR) as an inflammatory marker to distinguish major depression from healthy controls, but the SII cannot be used as a marker to distinguish depression patients with and without suicide attempts (35).In contrast, Cui et al. divided patients into those with moderate or severe depression and those without moderate or severe depression according to the Hamilton score and found that the SII>540.78 was a risk factor for moderate/major depression (36). Therefore, the correlation between the severity of depression and the SII levels remains controversial.

In our study, patients were divided into those with severe depression and those with mild to moderate depression. The results revealed a positive correlation between the level of SII and the severity of depression, and the SII can serve as an auxiliary diagnostic indicator for identifying major depression among the population with depression. This research project had a relatively large sample size and was based on hospitalized patients, with complete, accurate statistical data and high credibility. In addition, this study also included multiangle stratified analyses and sensitivity analysis to further verify the robustness of the association between the SII and the severity of depression. Overall, the SII was closely related to depression, which indicates that there is an urgent need to explore the potential underlying biological mechanism to conduct translational medicine research and apply inflammatory indicators such as the SII to the clinical diagnosis and treatment of mental diseases as soon as possible.

However, in the present study, we did not verify the biological mechanisms underlying the association between the SII and the risk of major depression. After reviewing the literature, we found that the following biopathological and physiological mechanisms might be involved. First, Rhie et al. discovered that various peripheral immune cells, including neutrophils, monocytes, and lymphocytes, release pro-inflammatory cytokines such as IL-1, IL-6, TNF-α, and interferon-γ (IFN-γ) to reach the brain. These cytokines can activate astrocytes and microglia to regulate the brain regions related to mood regulation, causing neuroinflammation and promoting excitotoxicity (increased glutamate content), which leads to brain cell damage and increased risk of developing depression (37). Moreover, a research by Dantzer et al. noted that the basic communication mechanisms between immune cells and the central nervous system include the impact of neuroendocrine hormones on the immune system, the innervation of lymphoid organs by the sympathetic nervous system, and the regulatory role of cytokines in the hypothalamus–pituitary–adrenal axis, which explains the influence of the immune system on brain functions and mental disorders (38). Furthermore, abnormalities in peripheral CD4^+^ T cells have a key mediating role in mood disorders, and severe mitochondrial fission in CD4^+^ T cells can trigger purine metabolism disorders, which further lead to various behavioral abnormalities including anxiety, depression, and social disorders (39). In addition, inflammation can induce the release of serotonin and other pro-inflammatory molecules from the dense granules within activated platelets, playing an important role in the development of depression (40). Future studies should further explore the potential biological mechanisms underlying the relationship between the SII and the risk of developing depression.

Despite the exciting results of this study, several limitations inevitably exist. First, because this study was a retrospective observational study, it cannot prove a causal association between the SII and the risk of major depressive disorder. In future studies, some genetic association studies should be carried out to further reveal the causal relationships among these factors. Second, this was a single central study from China, which might not be sufficient for extrapolating these results to other ethnicities and populations; thus, more multicenter prospective studies are needed to explore this association further. Third, although the study included more baseline data, it inevitably missed some important parameters that could have influenced the results, such as genetic predisposition, dietary factors, environmental factors, and lifestyle. Therefore, in future research, we will address this problem further. Finally, although the SII reflects the immune and inflammatory status of the whole body, it may not adequately assess the inflammatory status of the body, and some traditional speech indicators such as high-sensitivity CRP, IL-6, IL-8, IL-10, and IFN-γ may also outperform the SII in predicting the severity of depression. However, we lacked these variables in this study, so the strengths and weaknesses of their predictive value for depression severity are unknown. In future studies, we will assess the predictive value of multiple inflammatory markers for depression risk and severity as comprehensively and systematically as possible.

## 5 Conclusion

This retrospective study, which was based on data from a single-center medical center in China, confirmed a strong association between the SII and the risk of major depressive disorder, which not only expands the field of SII research, but also suggests that persistent sensitivity to excessively high levels of the SII in patients with depression should be maintained to prevent the excessive incidence of major depression and poor prognosis. Future studies should further explore the associations between the SII and the risk and severity of depression in more larger, multicenter prospective clinical studies and further explore the underlying biological mechanism in more rigorous cell and animal experiments.

## Data Availability

The raw data supporting the conclusions of this article will be made available by the authors, without undue reservation.
