# Supplementary material for: The correlation between systemic immune-inflammation index and major depression in patients with depression
Source: Front Psychiatry. 2025 May 8;16:1580151. doi: 10.3389/fpsyt.2025.1580151 (PMC12095310; doi:10.3389/fpsyt.2025.1580151)

***Supplementary Material***

1. **Supplementary Figure**

**Supplementary Figure 1** The predictive value of SII for depression severity.


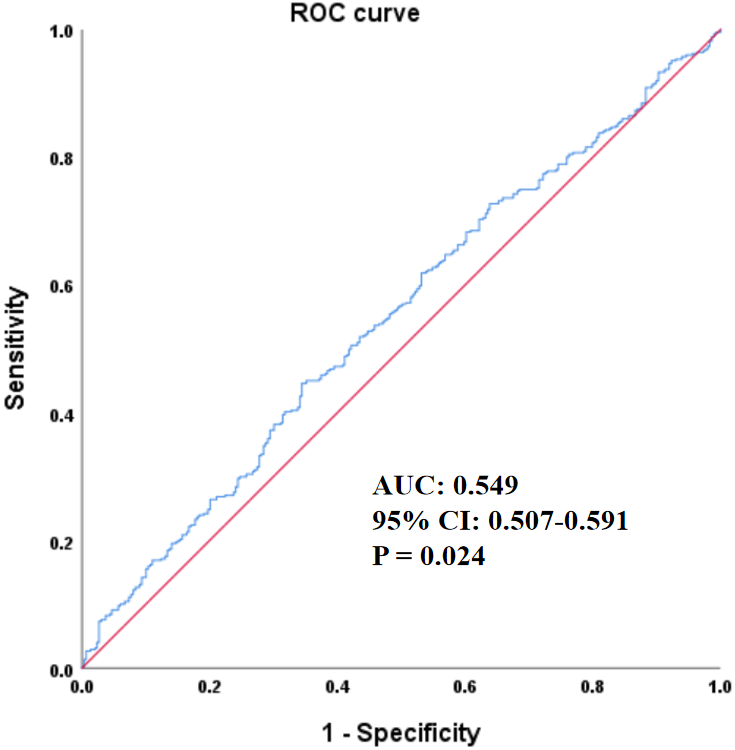

Supplement: Supplementary file 1 [file DataSheet1.docx]
