# Supplementary material for: The correlation between systemic immune-inflammation index and major depression in patients with depression
Source: Front Psychiatry. 2025 May 8;16:1580151. doi: 10.3389/fpsyt.2025.1580151 (PMC12095310; doi:10.3389/fpsyt.2025.1580151)
Supplement: Supplementary file 2 [file Table1.docx]

***Supplementary Material***

**2.Supplementary Data and Table**

In addition, a total of 200 people who received physical examination in Xiamen Xianyue Hospital from January 2022 to December 2023 , over 30 years old and without diagnosis of depression were selected as the health group. 750 depressed patients served as the depression group.

As shown in Supplementary Table 1, Compared with healthy group, depressed group were older and more female, lymphocyte and hemoglobin levels were lower, and SII levels were higher (P < 0.05).

| **Supplementary Table 1** Baseline characteristics of healthy group and depression group. | | | |
| --- | --- | --- | --- |
| Variables | Depression | Healthy | P value |
| N | 750 | 200 |  |
| Age, year | 51.65 ± 12.99 | 44.26 ± 9.11 | < 0.001 |
| Male, n (%) | 223 (29.7) | 87 (43.5) | < 0.001 |
| WBC, x10^9^/L | 6.40 ± 1.83 | 6.50 ± 1.74 | 0.459 |
| Neutrophil counts, x10^9^/L | 3.77 ± 1.52 | 3.69 ± 1.29 | 0.475 |
| Lymphocyte counts, x10^9^/L | 1.98 ± 0.62 | 2.14 ± 0.68 | 0.002 |
| Monocyte counts, x10^9^/L | 0.47 ± 0.18 | 0.48 ± 0.15 | 0.841 |
| Hemoglobin, g/L | 131.13 ± 14.86 | 138.18 ± 15.04 | < 0.001 |
| Platelets, x10^9^/L | 248.03 ± 63.63 | 239.80 ± 58.26 | 0.098 |
| SII, x10^9^/L | 428.91 (307.84, 646.45) | 385.89 (293.20, 531.63) | 0.007 |
